# Optical Coherence Tomography and Coronary Dissection: Precious Tool or Useless Surplus?

**DOI:** 10.3389/fcvm.2022.822998

**Published:** 2022-04-01

**Authors:** Lucia Barbieri, Andrea D’Errico, Carlo Avallone, Domitilla Gentile, Giovanni Provenzale, Giulio Guagliumi, Gabriele Tumminello, Stefano Carugo

**Affiliations:** ^1^Cardiology Unit, Fondazione IRCCS Ca’ Granda Ospedale Maggiore Policlinico, Department of Clinical Science and Community Health, University of Milan, Milan, Italy; ^2^Department of Medicine, Ospedale Papa Giovanni XXIII, Bergamo, Italy

**Keywords:** spontaneous coronary dissection, optical coherence tomography, interventional tools, intravascular ultrasound (IVUS), coronary vessel

## Abstract

Spontaneous coronary artery dissection (SCAD) is a rare clinical condition, but frequently manifested as acute myocardial infarction. In this particular setting, in recent years, optical coherence tomography (OCT) has been established as a possible diagnostic method due to the high spatial resolution (10–20 μm), which can visualize the different layers of coronary vessels. OCT can better analyze the “binary” or double lumen morphology, typical of this entity. Furthermore, it can identify the entrance breach and the circumferential and longitudinal extension of the lesion. However, we have to emphasize that this technique is not free from complications. OCT could further aggravate a dissection or exacerbate a new intimal tear. Therefore, the use of OCT in the evaluation of SCAD should be defined by balancing the diagnostic benefits versus procedural risks. Moreover, we underline that as SCAD is a rare condition and OCT is a recently introduced technique in clinical practice, limited data is available in literature.

## Spontaneous Coronary Artery Dissection

Spontaneous coronary artery dissection (SCAD), routinely considered a peculiar cause of acute coronary syndrome (ACS) (1, 2), is currently being increasingly recognized. Clinical display may range from unstable angina to sudden cardiac death. According to the Fourth Universal definition of myocardial infarction (2018) (3), SCAD is labeled as a spontaneous, non-iatrogenic and non-traumatic rupture of the coronary artery wall, leading to the formation of a false lumen and intramural hematoma (IMH). Insufficient evidence exists on the real prevalence and incidence of SCAD, as it is often underdiagnosed (4). Literature has shown a prevalence of 0.2–1.1% in coronary angiography in all patients presenting with ACS (5), yet a rate of up to 4.9% was stated in a recent manuscript where OCT was systematically performed in ACS (6). Recent perspectives on SCAD etiology focus on its multifactorial pathogenesis, including genetic factors, arteriopathies, connective tissue disorders, and systemic inflammatory diseases or vasculitis (7), but further investigation is needed in consideration of the extremely low observed frequency of such cases (8). Fibromuscular dysplasia (FMD) is a non-inflammatory and non-atherosclerotic arterial pathology, resulting in tortuosity and weakening of the arterial wall layers. FMD was correlated to SCAD (9), and most patients presenting with SCAD were angiographically diagnosed with FMD (1, 10). However, randomized controlled trials have not yet been driven and most evidences arouse from isolated case reports (8, 11, 12). Early evidence on potential mechanisms leading to SCAD originates from postmortem reports (13, 14). Two hypotheses have been highlighted to explain the pathophysiology. One is the “inside-out” hypothesis, which suggests that blood enters the subintimal space from the true lumen after development of an endothelial-intimal disruption or “flap” or intimal tear leading to an IMH. On the other hand, in the “outside-in” hypothesis, the hematoma arises *de novo* within the tunica media, because of disruption of traversing microvessels. Actual evidence favors the “outside-in” hypothesis, as most SCAD have no communication between true and false lumens (15–17), and intramural hematoma precedes development of intimal dissection (15). Although majority of SCADs are likely due to the latter mechanism, the phenotype may result from more than one pathophysiological mechanism. SCAD is associated with a small number of known genetic disorders (18, 19). A recent gene sequencing study showed that 3.5% of patients with SCAD had causal or likely pathogenic rare genetic variants, mostly in genes associated with other known disorders (e.g., vascular Ehlers-Danlos, Loeys-Dietz, and adult polycystic kidney disease) (20), while a monogenic basis for SCAD is less evident, although the finding of a common SCAD risk allele at the PHACTR1/EDN1 locus provides a rationale for genome-wide association studies (21).

### Clinical Presentation

The “typical” SCAD patient is a middle-aged woman lacking traditional cardiovascular risk factors with ACS symptoms (22). In some cases, it may be complicated by ventricular arrhythmias, cardiogenic shock, or sudden death (1). Sometimes, triggers have been proposed, such as emotional or physical stressors (1). However, it may also occur in other ACS scenarios, such as emotional stress with Takotsubo syndrome (23) or exercise and atherosclerotic plaque rupture (24). Accurate diagnosis is critical, as management of SCAD differs from atherosclerotic ACS, but also challenging, continuing to rely upon recognition of characteristic features on invasive angiography (25). SCAD occurs most commonly in the left anterior descending coronary artery (LAD) and in mid to distal coronary segments (26, 27). The Yip-Saw classification was developed to aid in diagnostic pattern recognition of SCAD and divides angiographic features into three types as follows (28):

– Type 1 (25%): typical contrast staining of the arterial wall is depicted along with a visible multiple radiolucent lumen; it is considered pathognomonic.
– Type 2 (70%): diffuse stenosis of varying severity with dissection and/or wall hematoma producing smooth coronary artery narrowing. It may be further classified into type 2A (normal arterial segment distal to the dissection) and type 2B (dissection extends to the distal tip of the coronary artery) (4).
– Type 3 (5%): variably long and diffuse stenosis that mimics atherosclerosis. Intracoronary imaging with optical coherence tomography (OCT)/intravascular ultrasound (IVUS) may clarify these cases (29).

This classification is limited by focusing essentially on the most common angiographic presentations. Recently, a modification has been proposed to add SCAD type 4 representing a vessel occlusion that does not meet the criteria for types 1–3 (30). Increased tortuosity of coronary vessels has been described in SCAD (31, 32). Intramural hematoma in patients with SCAD is frequently bounded at its proximal and distal extent by branch points (24). The differential diagnosis between SCAD and atheroma could be challenging due to a frequent overlap of angiographic findings (33).

## Physical and Technical Principles of Optical Coherence Tomography in Coronary Assessment

**FIGURE 1:**
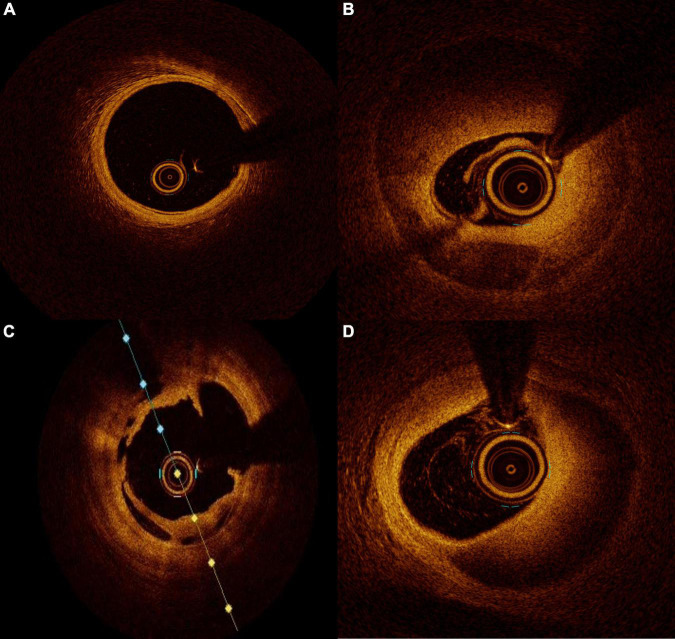
Intraluminal images seen on optical coherence tomography (OCT). **(A)** normal coronary artery, **(B)** coronary dissection with double lumen view, **(C)** intracoronary dissection flap, and **(D)** intramural hematoma.

Optical coherence tomography is an imaging modality that can be used in interventional cardiology to assess the severity and extent of atherosclerotic plaques in coronary arteries (34, 35). Furthermore, it allows a precise visualization of the different layers (intima, media, and adventitia) of the vessel wall as well as the histological examination, making this technique useful in the evaluation of coronary artery anatomy (36–38). This innovative endovascular imaging modality uses near-infrared light emission (in the 1,300 nm range) to generate high-resolution images (range 15–20 mm) and high sampling rate tomographic sections of coronary arteries in real time. Light beam that illuminates the inside of the vessel rotates rapidly, allowing a longitudinal scan of most of the coronary artery (7.5–15 cm) in a few seconds (2–3.5 s) (35). Fare clic o toccare qui per immettere il testo. There are two types of OCT systems: time domain (TD-OCT) and frequency domain (FD-OCT), which are, respectively, first and second generation. An essential requirement for image acquisition using TD-OCT was the absence of blood in the coronary vessel during the entire acquisition through balloon occlusion in proximal vessel (occlusive approach) or flushing with a large amount of fluid (non-occlusive approach). Furthermore, low frame rate (15.6 f/s) and low pullback speed (1–2 mm/s), beyond the risk of endothelial injury and myocardial ischemia and ventricular fibrillation, currently limit its use (39, 40). Since 2008, new generation OCT (frequency-domain, also called Fourier-domain) have been available for clinical use, which is more faster (frame rate 100 f/s, pullback speed 20 mm/s) and pullback images are acquired during flushing with low dose of contrast media (40). The patient is routinely anticoagulated with heparin, and intracoronary nitroglycerine is administered to minimize the potential catheter-induced vasospasm (41). OCT assessment is then performed by inserting a small catheter (2.7 French) over a guide wire, using standard guide catheters (6F or more). Next, the catheter-imaging tip is automatically pulled back to scan the coronary artery. The scan rate is approximately 20 mm/s and image capture rate is 100 f/s over 2.7 s, allowing imaging of 72 mm of vessel during a single run (36, 37, 41). The simultaneous injection of contrast medium throughout the duration of the pullback allows the light to interact with the surrounding vascular structures without any interference. The total amount of contrast medium required is 10–12 ml to scan a 50 mm long arterial segment (36, 41). The optic wire can send light to the coronary wall and record the reflection using low-coherence interferometry, by measuring the echo time delay and intensity of the light reflected from the structures in the tissue to generate cross-sectional images (37, 42). The intensity and attenuation of the recaptured optical signal are the basis of the tissue characterization conducted by OCT. Fare clic o toccare qui per immettere il testo. A normal coronary vessel segment appears on OCT as a trilaminar structure (Figure 1A). The *intima* appears as a hyper-reflective, homogeneous layer as compared to the lumen. The *media* appears as a low-signal layer, which surrounds the vessel, while the *adventitia* and external elastic lamina appear as a homogeneously irregular external network (34, 43).

## Optical Coherence Tomography Versus Intravascular Ultrasound

Nowadays, the two main protagonists for intracoronary imaging are IVUS and OCT, where IVUS is the most diffused and friendly technique for interventional cardiologists due to its presence over decades in clinical practice (44). Both intracoronary imaging methods, IVUS and OCT, allow real-time tomographic assessment of vessel size, lumen area, plaque composition, and volume, as well as stent coverage and expansion, as stated by 2020 ESC/EACTS guidelines for the management of ACS (45). IVUS and OCT have specific peculiarities that make each one more specific for different situations, and no one method is the definitive and preferable method in every condition. OCT has the highest resolution (axial 12–18 μm and lateral 20–90 μm), about ten times greater than that of IVUS (axial 150–200 μm and lateral 150–300 μm) (37). The higher resolution of OCT enables more detailed evaluation at the endoluminal level (46). OCT detects fine details, below the resolution of IVUS, clearly defining conditions, such as culprit lesion identification, plaque rupture vs. erosion or vulnerable plaque visualization, and tissue coverage of stent struts, to which IVUS may be blind (47, 48). OCT reaches a semi-histological assessment making possible the identification of macrophage accumulation into the plaque (49). The possible online three-dimensional (3D) reconstruction is instead one optional advantage of OCT (50). Due to the necessity of contrast media injection for blood clearance and due to the complexity of run acquisition, OCT may not be used to monitor every passage of an interventional procedure. On the other side, IVUS has higher tissue penetration depth (4–8 mm, while OCT has 1–3 mm), impaired by calcified lesions (37) which are conversely penetrated by OCT (51). IVUS well characterizes fibrous lesions and lipid pools (52). IVUS scan may be repeated several times during the same procedure and may be used to check the correct progression step-by-step.

## Discussion

Due to the inability to visualize the different layers of the coronary wall, angiography should not be considered the gold standard to investigate SCAD. Angiography needs to be integrated by an intra-coronary imaging technique. OCT has an evident advantage over IVUS in the diagnosis of SCAD because of its superior spatial resolution and its ability to identify intramural hematoma, endothelial tears, or entry sites of dissection. The only concerns about OCT use in the SCAD diagnostic process is the possibility of progression of false lumen due to the contrast injection required for imaging acquisition. The contrast flush may generate a hydraulic injury propagating the dissection or expanding an intramural hematoma up to compromise the artery distal flow. This fear is more theoretical than practical. In small series, it has been evidenced that OCT acquisition during SCAD is safe and feasible (53). The contrast injection for OCT acquisition is not more harmful than the usual one for angiography imaging acquisition. For both, a cautious and delicate engagement of guiding catheter is crucial, and in case of staining of contrast along the artery profile with a low or absent run off, any injection for any reason is not recommended. As previously reported, angiography has intrinsic limitations in SCAD identification but may has also an overestimation of the problem. Alfonso and colleagues evaluated a large prospective series of SCAD diagnosed with traditional angiography using OCT. SCAD was confirmed by OCT only in 11 patients out of 17. In the remaining six patients, OCT ruled out the presence of SCAD by finding severe atherosclerosis, calcified lesions, or intracoronary thrombus mimicking SCAD (54). Once SCAD has been diagnosed, OCT is fundamental for its management. Considering that the healing process of the vessel wall is most of the time an autonomous process and the natural history of SCAD most of the time involves without any intervention, the conservative strategy is the most appropriate in majority of the cases (54). In this context, OCT can define the precise length of affected wall, the presence of an intramural hematoma, the degree of luminal compromission, and the thickness of the dissected tear (Figures 1B–D). All these information are essential for decision and planning an eventual percutaneous coronary intervention (PCI). In case of unavailability of OCT, IVUS may be considered a useful surrogate, but because of its relatively low spatial resolution it may not clarify all the questions regarding an SCAD. Nowadays, there are few evidence-based data for the management of SCAD, but based on registries, little prospective series, and expert opinion, PCI should be reserved to patients with proximal vessels dissection and ongoing ischemia or reduced/no coronary thrombolysis in myocardial infarction (TIMI) flow. In these cases where treatment is mandatory, angiography may overestimate the coronary segment needed to be treated. Moreover, OCT identifies the real reference diameters and the length of segment to be treated, thereby preventing possible complications. The incorrect stent sizing or deployment may result in intramural hematoma expansion or dissection rim advancement with a consequent degradation of coronary flow with the necessity of further stent deployment. Furthermore, in rare situations where IMH may bring to an overestimation of vessel dimensions at angiography, the incorrect stent sizing may get up to vessel wall rupture. To avoid the so-called “geographical mismatch,” the OCT co-registration technique is a useful tool, especially in this context (55). In conclusion, the use of an imaging modality to evaluate the vessel wall in a coronary pathology, as in SCAD, to guide the interventional cardiologist from diagnosis to the PCI planning, passing through the decision-making process must be considered the gold standard nowadays.

## Author Contributions

LB, GT, AD’E, and DG: conceptualization and data collection. AD’E, CA, DG, and GP: data collection and editing the manuscript. LB, GT, GG, and SC: editing, reviewing, and supervision of the manuscript. All authors contributed to the article and approved the submitted version.

## Conflict of Interest

The authors declare that the research was conducted in the absence of any commercial or financial relationships that could be construed as a potential conflict of interest.

## Publisher’s Note

All claims expressed in this article are solely those of the authors and do not necessarily represent those of their affiliated organizations, or those of the publisher, the editors and the reviewers. Any product that may be evaluated in this article, or claim that may be made by its manufacturer, is not guaranteed or endorsed by the publisher.
